# Lipid metabolism-associated metabolites on cardiovascular diseases: a two-sample Mendelian randomized study

**DOI:** 10.3389/fcvm.2025.1445732

**Published:** 2025-03-28

**Authors:** Jia-Le Chen, Xin-Yi Lu, Dao-Zhen Chen, Yu Chen

**Affiliations:** ^1^ Hospital Infection Management Section, Wujin Affiliated Hospital of Nanjing University of Traditional Chinese Medicine, Changzhou, Jiangsu, China; ^2^ Wuxi Maternal and Child Health Hospital, Wuxi School of Medicine, Jiangnan University, Wuxi, Jiangsu, China; ^3^ Wuxi Medical Center, Nanjing Medical University, Wuxi, Jiangsu, China

**Keywords:** plasma metabolites, cardiovascular disease, glycolipid metabolic disorders, Mendelian randomization, single-nucleotide polymorphisms, risk factors

## Abstract

**Background:**

There is a growing body of evidence indicating that metabolites are associated with an increased risk of cardiovascular diseases (CVDs), the underlying causality of these associations remains largely unchallenged. Given the inherent difficulty in establishing causality using epidemiological data, we employed the technique of Mendelian randomization to investigate the potential role of plasma metabolite factors in influencing the risk of CVDs.

**Methods:**

The exposure was based on 1,400 plasma metabolites, and outcomes involved four CVD datasets from public databases. Initial causality was assessed by inverse variance weighting (IVW), followed by sensitivity analyses using MR-Egger regression, weighted median, and Multiple Effectiveness Residual Sums and Outliers (MR-PRESSO) method. Potential heterogeneity and multivalence were assessed using the MR-Egger intercept and Cochran's Q statistic. After Bonferroni correction, causal associations were found to be significant with *p*-values less than 0.05. All statistical analyses were rigorously executed in R software.

**Results:**

Our findings identified causal relationships between 15 metabolites and cardiovascular disease. Of these, 4 were associated with AA (aortic aneurysm), 7 with atrial fibrillation and flutter, 2 with HF (heart failure), and 3 with stroke.

**Conclusion:**

This is the first systematic mendelian randomization analysis using genome-wide data to assess the causal relationship between serum metabolites and different cardiovascular diseases, providing preliminary evidence for the impact of lipid metabolism disorders on cardiovascular disease risk.

## 1 Introduction

Cardiovascular diseases (CVDs) represent a heterogeneous and complex group of disorders arising from various molecular events (1). Despite significant advances in understanding the pathophysiology of cardiovascular diseases, these diseases remain the leading cause of death worldwide (2, 3). Effective management of CVDs is thus crucial for reducing the global health burden.

In recent years, glycolipid metabolism disorders have garnered significant attention for their profound impact on vascular health, contributing to the development of cardiovascular and cerebrovascular diseases. These disorders alter lipid and carbohydrate metabolism, leading to endothelial dysfunction, inflammation, and atherosclerosis (4, 5). The associated pathophysiological mechanisms are crucial in disease progression, initiating detrimental effects such as increased oxidative stress, inflammation, and plaque accumulation in blood vessels. Metabolic products like lipids, sugars, and related compounds exacerbate disease progression by promoting lipid accumulation, insulin resistance, and vascular calcification. Therapeutics targeting glycolipid metabolism disorders offer promising treatments for these diseases by modulating metabolic pathways to restore balance, reduce inflammation, and prevent plaque formation. Developing and refining such therapies could significantly improve patient outcomes and reduce the global burden of cardiovascular diseases.

Circulating metabolites are intermediates or end products of metabolic activities in organisms, belonging to small molecular compounds (molecular weight usually less than 1 kDa), mainly including amino acids and their derivatives, carbohydrates, lipids, and xenobiotics-related metabolites (6). A total of 48 metabolites are frequently utilized in the investigation of physiological and pathophysiological processes (7, 8). Deidda et al. (9) demonstrated the correlation between metabolic compounds, including 2-hydroxybutyrate, glycine, methylmalonate, and myo-inositol, and the terminal complications associated with CVDs. However, the specific pathophysiological mechanisms through which metabolites influence CVDs remain unclear. Therefore, the study of metabolites associated with CVDs not only contributes to the understanding of the biological mechanisms of cardiovascular disease, but also aids in the early screening and prevention of the disease.

Current metabolite studies are limited to a few metabolites and are often constrained by the inherent shortcomings of traditional epidemiological studies, such as insufficient sample sizes, the influence of confounding variables, and the risk of causal inversion. The MR method provides a more reliable analytical framework for causal inferences between exposures and outcomes by exploiting the natural random assignment mechanism of genetic variation (10). Compared to randomized controlled trials (RCTs), MR method can mitigate chance bias and avoid confounding and misleading associations between modifiable exposures and diseases in observational studies. Moreover, MR method typically demands fewer human resources and less follow-up (11). Given stringent experimental conditions and resource constraints in large-scale RCTs, MR method has become a valuable way to study biological mechanisms. Our study fills this gap by systematically investigating the causal relationship between a comprehensive set of plasma metabolites and multiple cardiovascular diseases using a two-sample MR method and genome-wide data.

## 2 Materials and methods

### 2.1 Study design

**Figure 1:**
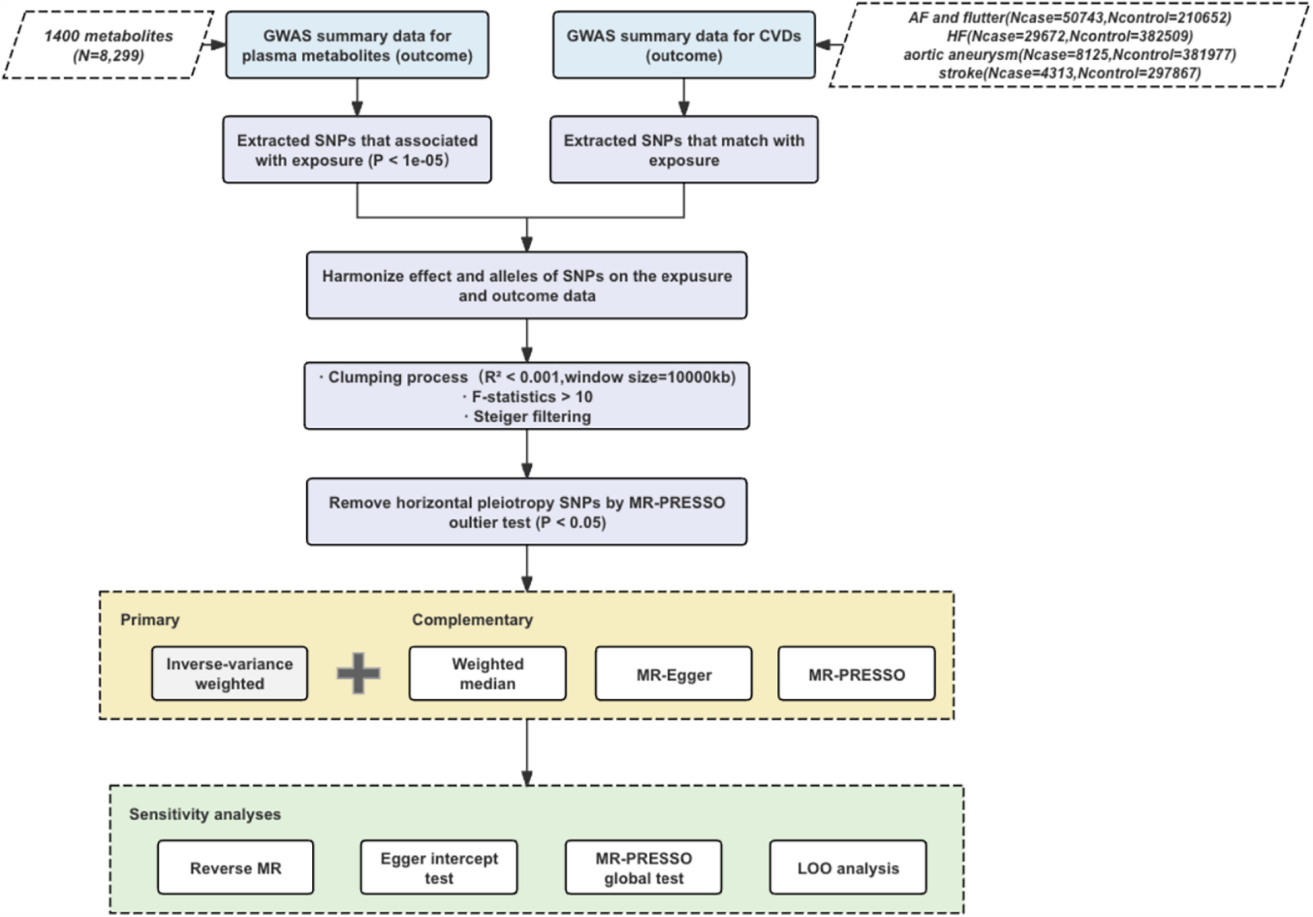
The following terms are used throughout this study.

The study workflow is illustrated in Figure 1. In this study, the plasma concentration of each metabolite was designated as the exposure variable, and the risk of each CVD was defined as the outcome variable. Single nucleotide polymorphisms (SNPs) that demonstrated significant associations with the exposure variables were employed as instrumental variables (IVs). To ensure the robustness of Mendelian randomization studies the following principles should be followed: strong correlation between instrumental and exposure variables; independence of instrumental variables; exclusivity assumptions; and independence of outcome variables (12, 13). Linkage disequilibrium (LD) clustering minimizes multicollinearity by reducing the number of highly correlated SNPs, enhances the validity of statistical tests, and eliminates potential bias. The F-statistic was evaluated to confirm the existence of differences and their statistical significance, and Steiger's test was conducted to verify the validity of instrumental variables. Egger regression and MR-PRESSO methods jointly tackle the problems of multiplicity and heteroscedasticity in MR studies through different statistical strategies (intercept term correction vs. outlier rejection). Joint application of these two methods can identify data bias more comprehensively and improve the accuracy and reliability of causal inference. Leave-one-out (LOO) analyses were performed to assess the effects of individual instrumental variables. In addition, reverse MR analyses were conducted to explore the possibility of reverse causality (14–16).

### 2.2 GWAS data of serum metabolites

The most comprehensive analysis of human metabolites to date involves a genome-wide dataset containing 1,400 metabolites (17), and its complete summary statistics are publicly available through the GWAS Catalog (https://www.ebi.ac.uk/gwas/). A total of 8,299 unrelated European subjects from the Canadian Longitudinal Study of Aging (CLSA) cohort with approximately 150,000 SNPs were included in this genome-wide association analysis (GWAS). Out of the 1,091 plasma metabolites assayed, 850 were identified and classified into eight super pathways (namely, lipid, amino acid, xenobiotics, nucleotide, cofactor and vitamins, carbohydrate, peptide, and energy), while the remaining 241 were labeled as unknown or “partially” characterized compounds.

### 2.3 GWAS data for cardiovascular disease

GWAS summary data on aortic aneurysm (8,125 cases and 381,977 controls), atrial fibrillation and flutter (50,743 cases and 210,652 controls), heart failure (29,672 cases and 382,509 controls), stroke (4,313 cases and 297,867 controls) were obtained from results of the GWAS on the FinnGen consortium R10 shown in Table 1.

**Table 1:** Characteristics of the summary datasets for adverse pregnancy outcomes.

Adverse pregnancy outcomes	Sample size	Case	Control
All			
Aortic aneurysm	3,90,102	8,125	3,81,977
Atrial fibrillation and flutter	2,61,395	50,743	2,10,652
Heart failure	4,12,181	29,672	3,82,509
Stroke	3,02,180	4,313	2,97,867

### 2.4 Selection of instrumental variables

For each metabolite, minor allele frequencies (MAF) greater than 0.05 in the 1,000 Genomes Project and GWAS with CVDs showing non-allelic SNPs were used for IV selection. SNPs independently associated with plasma metabolites at a significance level of *P* < 5 × 10^−8^ were identified using LD clumping with a window size of 1,000 kb, ensuring pairwise LD r² values were less than 0.001 (18). For each metabolite, we calculated the proportion of variance in its plasma level explained by each IV using the *R*² formula: *R*² = [2*β*² × EAF × (1–EAF)]/[2*β*² × EAF × (1–EAF) + 2N × EAF × (1–EAF) × SE²]. Additionally, the strength of each IV was assessed using the F-statistic, calculated as F = [*R*² × (N–2)]/(1–*R*²), where EAF is the effect allele frequency, β is the effect size, SE is the standard error, and N is the sample size. In these equations, EAF stands for the frequency of the effect allele, while β symbolizes the magnitude of the association between the SNP and the metabolite (i.e., the effect size), and SE indicates the standard error of this association. N represents the total number of samples in the metabolite GWAS (19). Metabolites retaining at least three valid instrumental variables will be considered as fulfilling the conditions for MR analysis after excluding weak IV (F-statistic <10) and outliers identified by MR-PRESSO test (*P* < 0.05).

### 2.5 MR analysis

The primary approach utilized for MR analyses was the inverse variance-weighted (IVW) method. IVW estimates, which are derived from a comprehensive analysis of Wald ratios for all genetic variants, are known to account for the assumption of no horizontal pleiotropy across all SNPs (18). To control for the incidence of type I errors, we applied a Bonferroni correction to the analyzed results for each CVD outcome. To ensure the robustness of the findings, we also performed additional analyses using three other MR methods. Specifically, we used the weighted median method to counter the possible bias of the strong assumption that all IVs are valid in the IVW method (20). Also, to identify and correct for pleiotropic effects caused by genetic variation that affects both exposure factors and outcome variables, we used the MR-Egger method of adjustment (16). Furthermore, we employed the MR-PRESSO method (15) to identify and correct for the effects of outliers on MR estimates.

### 2.6 Complementary, sensitivity, and reverse MR analyses

To further scrutinize the robustness of the significant associations identified through IVW method, we undertook a comprehensive set of complementary and sensitivity analyses. Specifically, we conducted heterogeneity tests to verify the appropriateness of IVs, applied the Egger intercept test and the MRPRESSO global test to evaluate potential horizontal pleiotropy, and performed leave-one-out (LOO) analyses to detect any overly influential IVs (21). To explore possible reverse causality in the significant associations found, we performed reverse MR analyses with CVD as the exposure variable and metabolites as the outcome variable. The main objective of the reverse Mendelian randomization analysis is to investigate whether the association between “cause” and “effect” still holds when they are interchanged in the traditional sense, which helps to understand whether CVD may be caused by changes in certain metabolites. Owing to the considerably larger sample size of the CVD GWAS, we employed stricter criteria for selecting SNPs. Specifically, we chose SNPs that were independently associated with CVDs (pairwise linkage disequilibrium *r*^2^ < 0.001 within a 10,000 kb window) at a genome-wide significance threshold of *P* < 0.05 to serve as IVs. In subsequent analyses, we considered correlations with *P* < 0.05 estimated by IVW method as statistically significant.

### 2.7 Power calculation

The statistical strength of the MR estimates was assessed by means of the R software. Power calculations were performed at a type I error rate of 0.05, taking into account parameters such as the *R*^2^ of IVs, the proportion of GWAS cases with cardiovascular disease, and OR for MR analysis using the IVW method.

### 2.8 Statistical analysis

The statistical analyses were conducted using R software version 4.3.3. For the MR analyses, we utilized the R packages T*woSampleMR* (version 0.6.1) and *MR-PRESSO* (version 1.0).

## 3 Results

### 3.1 Genetic IVs

Of the 1,400 metabolites, 1,299 independent SNP component instrumental variables were collected using LD clumping criteria of pairwise *r*^2^ < 0.001 within a 10,000 kb window and a significance threshold of *P* < 5 × 10^−8^. Following a rigorous screening process to exclude weak IVs and outliers, the subsequent MR analyses were conducted with a refined set of metabolites. This screening process utilized various criteria, including F-statistics <10 and Steiger's test, in addition to applying the MR-PRESSO outlier test *P* < 0.05. The specific details regarding IVs selected for the subsequent MR analyses are provided in Supplementary Table S2.

### 3.2 Effects of genetically determined metabolites on CVDs

To enhance the interpretability of metabolic changes, we excluded 309 metabolite ratios and retained 1,091 individual metabolites for analysis. The Bonferroni-corrected *P*-values represent the raw *P*-values adjusted for multiple comparisons, thereby minimizing the likelihood of false-positive findings. Utilizing IVW method, we identified 15 causal associations with a Bonferroni-corrected *P* < 0.05. These associations involved eight metabolites, eight of which belonged to the lipid metabolism pathway, one to the peptide pathway, and one to the xenobiotic pathway (Table 2).

**Table 2:** Significant associations identified in primary MR analyses using the inverse-variance weighted (IVW) method.

Metabolites	Categories	Number of IVs	IVW			MR Egger		Weighted median		MR PRESSO	
			OR(95% CI)	*P*	*P*_bonfer_	OR(95% CI)	*P*	OR(95% CI)	*P*	OR(95% CI)	*P*
Aortic aneurysm											
Octadecanedioate	Lipid	4	1.25 (1.10–1.41)	0.0006	0.0120	1.02 (0.66–1.58)	0.93	1.25 (1.07–1.46)	0.005	1.25 (1.01–1.53)	0.036
Palmitoyl sphingomyelin	Lipid	4	0.72 (0.61–0.86)	0.0003	0.0049	0.58 (0.40–0.86)	0.11	0.69 (0.55–0.86)	0.001	0.72 (0.55–0.96)	0.023
Sphingomyelin	Lipid	4	1.19 (1.08–1.32)	0.0008	0.0144	2.15 (0.87–5.32)	0.24	1.22 (1.07–1.39)	0.003	1.19 (1.02–1.40)	0.031
N-acetylglutamine	Amino Acid	4	0.92 (0.87–0.97)	0.0015	0.0281	0.96 (0.87–1.05)	0.45	0.92 (0.87–0.97)	0.004	0.92 (0.84–1.00)	0.050
Atrial fibrillation and flutter											
1-oleoylglycerol	Lipid	4	1.27 (1.13–1.43)	0.0001	0.0031	1.05 (0.59–1.85)	0.89	1.29 (1.14–1.47)	6.28 × 10^−5^	1.27 (1.02–1.58)	0.030
Nervonoylcarnitine	Lipid	5	0.90 (0.85–0.95)	0.0001	0.0031	0.94 (0.76–1.17)	0.62	0.91 (0.85–0.96)	0.0013	0.90 (0.84–0.96)	0.002
2-ketocaprylate	Amino Acid	4	0.88 (0.83–0.93)	0.0000	0.0002	0.89 (0.75–1.07)	0.34	0.87 (0.81–0.93)	2.54 × 10^−5^	0.88 (0.81–0.95)	0.002
X-21467	Unknown	6	1.07 (1.03–1.11)	0.0007	0.0228	1.07 (0.99–1.16)	0.18	1.07 (1.02–1.12)	0.0041	1.07 (1.01–1.13)	0.018
X-23659	Unknown	4	1.18 (1.07–1.30)	0.0008	0.0246	1.02 (0.85–1.23)	0.83	1.16 (1.05–1.28)	0.0031	1.18 (1.00–1.38)	0.044
N-acetyltyrosine	Amino Acid	5	0.95 (0.93–0.98)	0.0016	0.0495	0.99 (0.92–1.06)	0.74	0.96 (0.93–0.99)	0.0038	0.95 (0.92–0.99)	0.016
Heart failure											
Pregnenetriol sulfate	Lipid	6	1.11 (1.03–1.20)	0.0044	0.0437	1.08 (0.92–1.27)	0.42	1.10 (1.02–1.19)	0.0182	1.11 (1.03–1.20)	0.007
X-25422	Unknown	6	1.08 (1.02–1.14)	0.0048	0.0481	1.02 (0.89–1.18)	0.78	1.06 (0.99–1.12)	0.0871	1.08 (1.02–1.14)	0.009
Stroke											
Tetradecanedioate	Lipid	4	1.06 (1.02–1.10)	0.0010	0.0254	1.08 (1.02–1.15)	0.12	1.07 (1.03–1.11)	0.0007	1.06 (1.01–1.12)	0.031
Hexadecanedioate	Lipid	5	1.07 (1.03–1.10)	0.0002	0.0054	1.08 (1.03–1.14)	0.07	1.07 (1.03–1.11)	0.0002	1.07 (1.01–1.12)	0.011
X-21467	Unknown	6	1.06 (1.02–1.10)	0.0014	0.0366	1.04 (0.97–1.11)	0.37	1.05 (1.01–1.09)	0.0087	1.06 (1.01–1.11)	0.024

Specifically, for aortic aneurysm (AA), the associations included: octadecanedioate [odds ratio (OR) = 1.25, 95% confidence intervals CI 1.10–1.41]; palmitoyl sphingomyelin (OR = 0.72, 95% CI 0.61–0.86); sphingomyelin (OR = 1.19, 95% CI 1.08–1.32); N-acetylglutamine (OR = 0.92, 95% CI 0.87–0.97). For atrial fibrillation and flutter, the associations were: 1-oleoylglycerol (OR = 1.27, 95% CI 1.13–1.43); nervonoylcarnitine (OR = 0.90, 95% CI 0.85–0.95); 2-ketocaprylate (OR = 0.88, 95% CI 0.83–0.93); X-21467 (OR = 1.07, 95% CI 1.03–1.11); X-23659 (OR = 1.18, 95% CI 1.07–1.30); N-acetyltyrosine (OR = 0.95, 95% CI 0.93–0.98). For heart failure, the associations were: pregnenetriol sulfate (OR = 1.11, 95% CI 1.03–1.20); X-25422 (OR = 1.08, 95% CI 1.02–1.14). For stroke, the associations were: tetradecanedioate (OR = 1.06, 95% CI 1.02–1.10); hexadecanedioate (OR = 1.07, 95% CI 1.03–1.10); X-21467 (OR = 1.06, 95% CI 1.02–1.10).

### 3.3 Sensitivity analysis

**Figure 2:**
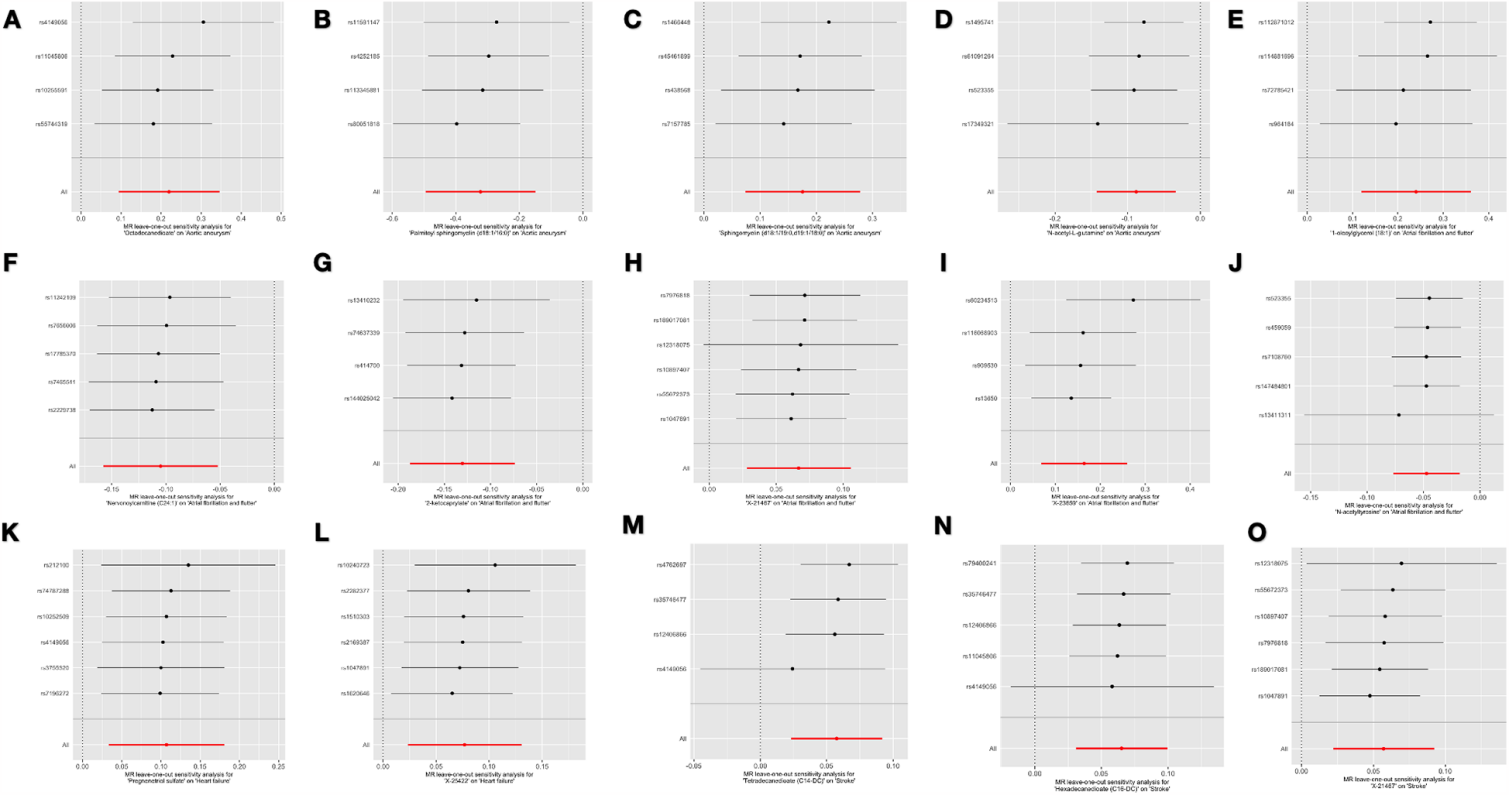
The funnel plot illustrates the influence of omitting a single SNP on the overall effect estimate and displays the instrumental variables (IVs) for each significant causal association between metabolites and CVDs. **(A)** Octadecanedioate on aortic aneurysm; **(B)** Palmitoyl sphingomyelin on aortic aneurysm; **(C)** Sphingomyelin on aortic aneurysm; **(D)** N-acetylglutamine on aortic aneurysm; **(E)** 1-oleoylglycerol on atrial fibrillation and flutter; **(F)** Nervonoylcarnitine on atrial fibrillation and flutter; **(G)** 2-ketocaprylate on atrial fibrillation and flutter; **(H)** X-21467 on atrial fibrillation and flutter; **(I)** X-23659 on atrial fibrillation and flutter; **(J)** N-acetyltyrosine on atrial fibrillation and flutter; **(K)** Pregnenetriol sulfate on heart failure; **(L)** X-25422 on heart failure; **(M)** Tetradecanedioate on stroke; **(N)** Hexadecanedioate on stroke; **(O)** X-21467 on stroke.

**Figure 3:**
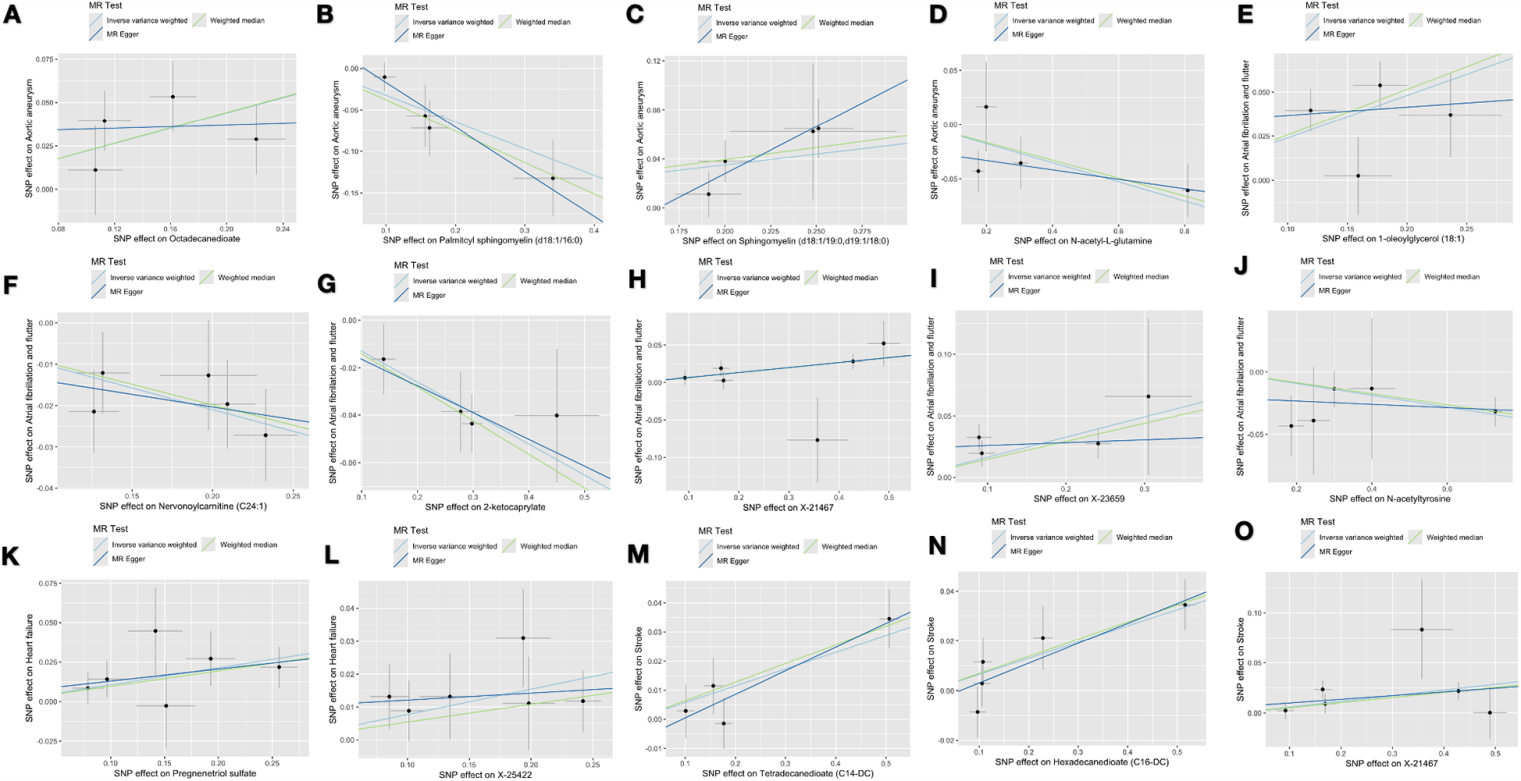
The scatter plots depict the genetic associations of 15 metabolites with the risk of 4 CVDs. **(A)** Octadecanedioate on aortic aneurysm; **(B)** Palmitoyl sphingomyelin on aortic aneurysm; **(C)** Sphingomyelin on aortic aneurysm; **(D)** N-acetylglutamine on aortic aneurysm; **(E)** 1-oleoylglycerol on atrial fibrillation and flutter; **(F)** Nervonoylcarnitine on atrial fibrillation and flutter; **(G)** 2-ketocaprylate on atrial fibrillation and flutter; **(H)** X-21467 on atrial fibrillation and flutter; **(I)** X-23659 on atrial fibrillation and flutter; **(J)** N-acetyltyrosine on atrial fibrillation and flutter; **(K)** Pregnenetriol sulfate on heart failure; **(L)** X-25422 on heart failure; **(M)** Tetradecanedioate on stroke; **(N)** Hexadecanedioate on stroke; **(O)** X-21467 on stroke.

Supplementary Table S3 lists the 18 significant associations identified using the inverse IVW method. Among these 18 associations, three metabolites exhibited different patterns of association when compared with the results of the other three MR methods (i.e., weighted median test, MR-Egger test, and MR-PRESSO test). To assess and mitigate potential horizontal multidirectionality in MR estimates, we performed sensitivity analyses and presented the results in Figure 2, which relates to 15 metabolite-cardiovascular disease pairs with significant causal associations. Overall, a causal relationship was considered robust if at least three additional MR methods were statistically significant (*P* < 0.05). Notably, 15 of these 15 associations had a *P* < 0.05 in at least two of the three supplemental MR analyses. The MR-Egger intercept term and the MRPRESSO global test indicated that only one association was affected by horizontal multidimensionality, namely, the effect of carnitine arachidonic acid on AF and flutter (for details, see Supplementary Table S3). In addition, to identify and exclude potential outliers and to assess the presence of horizontal multidirectionality among the identified metabolites, we also used scatter plots (Figure 3) and funnel plots (Figure 2). LOO analyses confirmed that none of the 15 associations were found to be dominated by any of the IVs (instrumental variables). Also, no significant heterogeneity among IVs was found in these 15 associations (*P* > 0.05 for heterogeneity). In the reverse MR analysis, 72 SNPs were selected as IVs for AA, AF and flutter, heart failure, and stroke, respectively. For detailed results of the sensitivity and multinomial analyses shown in Supplementary File S1: Supplementary Table S1.

## 4 Discussion

Studies have shown that disorders of lipid metabolism are an important threat to cardiovascular health, leading to endothelial dysfunction, inflammation, and atherosclerosis, which in turn have serious implications for vascular health. These metabolic disorders are closely associated with the onset and progression of CVD. Therefore, an in-depth understanding of the causal role of specific lipid metabolites in CVD will not only help to unravel the underlying biological mechanisms, but also provide critical information for identifying potential therapeutic targets for intervention.

The present study sought to explore the potential for causality between metabolites and CVDs by leveraging existing GWAS data and two-sample MR. To enhance the reliability of the findings, extensive sensitivity analyses were conducted, which involved the exclusion of potential confounders. This study represents a novel approach that combines metabolomics and genomics to systematically investigate the causal relationship between serum metabolites and multiple CVDs. Our findings identified causal relationships between 15 metabolites and cardiovascular disease. Of these, 4 were associated with AA, 6 with atrial fibrillation and flutter, 2 with heart failure, and 3 with stroke. These metabolites are hypothesized to function as biomarkers, capable of facilitating early screening and risk prediction for cardiovascular disease. In addition, interventions targeting these metabolites or their metabolic pathways may provide novel strategies for the prevention or treatment of cardiovascular disease. For example, modulating the levels of specific metabolites through diet, lifestyle changes, or pharmacological interventions could potentially reduce the risk of CVDs.

Numerous prior studies have demonstrated substantial interest in exploring the potential molecular mechanisms of metabolites in the pathogenesis of AA. Metabolic disorders may lead to metabolic diseases such as hypertension, hyperglycemia, and hyperlipidemia, which are risk factors for atherosclerosis (22). Atherosclerosis increases the risk of AA by weakening the aortic wall (23). In addition, metabolic disorders may affect the normal structure and function of the blood vessel wall, further promoting the formation of AA (24). We successfully identified that octadecanedioate and sphingomyelin are positively associated with the risk of AA, palmitoyl sphingomyelin (PSM) and N-acetylglutamine are negatively associated with the risk of AA. Sphingolipids and ceramides are associated with each other through the so-called “sphingomyelinase-ceramide pathway”, which is the product of sphingomyelin hydrolysis by sphingomyelinase (25). The active sphingomyelinase-ceramide pathway is pro-inflammatory, pro-oxidative and induces apoptosis, which may contribute to atherosclerosis, accelerate the aging process and increase the risk of cardiovascular events (26, 27).It was shown that sphingomyelin (SM) levels were significantly increased in bicuspid aortic valve-associated aneurysms (BAV-A) and tricuspid aortic valve-associated aortic dissections (TAV-Diss) samples, which may indicate that sphingomyelin inhibition of sphingomyelinase activity and the sphingomyelinase-ceramide pathway, leading to inhibition of tissue regeneration; a potential basis for the onset and progression of AA (28). Untargeted metabolomics analysis found 16 lipids and fatty acids metabolites associated with CVD risk in patients with diabetes, with PSM having the strongest association (29). Circulating palmitoyl sphingomyelin levels predict the 10-year increased risk of CVD death in Chinese adults (30). These findings are inconsistent with our conclusion that there is a negative association between PSM and AA. Studies have shown that Sphingosine-1-phosphate (S1P) activates STAT3 through the S1PR2/S1PR3 signaling pathway and promotes survivin expression, which inhibits macrophage apoptosis and plays a protective role against atherosclerosis (31). Moreover, S1P binding to S1PR1 protects the permeability integrity and barrier function of vascular endothelial cells and inhibits inflammation and cardiovascular disease (32). S1P is an important molecule in the sphingolipid metabolic pathway and may be associated with PSM. This literature provides evidence for a protective role of S1P and its receptor in cardiovascular disease, which may be related to the protective effects of PSM. The mechanism of action of these compounds involves complex biological processes, including lipid metabolism, inflammatory response, cell proliferation and migration.

Atrial flutter is less common and is often associated with or preceded by atrial fibrillation (AF) and flutter or occurs in an isolated pattern (33). The present study found that 1-oleoylglycerol was positively associated with the risk of AF and flutter, and that neuraminic acid (Neu5Ac) and 2-keto-3-hexanoylglycerol (2-KG) were negatively associated with the risk of AF and flutter. Unfortunately, there have been no studies of metabolites in AF and flutter. There are no direct studies linking 1-oleoylglycerol and cerylcarnitine to inflammation or oxidative stress, but given the important role of these metabolites in energy metabolism and fat metabolism, as well as the key roles of inflammation and oxidative stress in AF, it can be hypothesized that these metabolites may indirectly influence the levels of inflammation and oxidative stress by affecting energy metabolism and fat metabolism. For example, disturbances in fat metabolism may lead to an increase in inflammatory factors and oxidative stress in the body, thereby increasing the risk of AF and flutter (34). Our results suggest that N-acetylglutamine was negatively correlated in AA, AF and flutter, which was the sole metabolite found to be associated with multiple CVDs. N-acetylglutamine may be employed in the treatment of other diseases, including liver disease, kidney disease, and metabolic disorders. These diseases may be associated with or interact with CVDs. Furthermore, N-acetylglutamine is a precursor of glutathione, which is involved in the antioxidant process *in vivo* (35). Oxidative stress is a significant factor in the development and progression of CVDs. Consequently, any substance that enhances antioxidant capacity in the body may have a beneficial effect on CVDs (36).

Heart failure, stemming from underlying myocardial pathology, is a heterogeneous disease process with various etiologies and can be broadly classified into ischemic and nonischemic categories (37). The severity of heart failure is closely tied to alterations in cardiac energy metabolism. However, these metabolic changes are highly complex, influenced not only by the severity and type of heart failure but also by common comorbidities such as obesity and type 2 diabetes. In the failing heart, an energy deficit occurs primarily due to reduced mitochondrial oxidative capacity. To compensate, there is an increase in ATP production via glycolysis. Meanwhile, the relative contributions of different substrates to mitochondrial ATP production shift, characterized by decreased glucose and amino acid oxidation and increased ketone body oxidation. Cardiac fatty acid oxidation also varies depending on the type of heart failure, with either an increase or decrease observed (38). Our study suggests that pregnanetriol sulfate may be a risk factor for heart failure, but this finding warrants further validation.

We successfully identified tetradecanedioate and hexadecanedioate as positively associated with stroke risk. This finding is in complete agreement with the results of an untargeted serum metabolomics study published in 2019. That study identified two long-chain dicarboxylic acids, tetradecanedioate and hexadecanedioate, whose serum levels were highly correlated and were independently associated with incident ischemic stroke (IS), even after accounting for known risk factors (39). Studies conducted in experimental models have revealed possible physiological functions of hexadecanedioate, including hypolipidemic, anti-obesity and antidiabetogenic properties (40). Diabetes mellitus, dyslipidemia and obesity are known risk factors for stroke (41). Recently, blood pressure levels and all-cause mortality have been associated with BP levels in Europe (42). Experiments conducted in a hypertensive rat model demonstrated that oral administration of BP levels led to increased circulating metabolite levels and elevated blood pressure in Wistar Kyoto rats. Additionally, mesenteric resistance arteries from rats treated with BP levels exhibited a heightened contractile response to norepinephrine. This enhanced response may provide a potential explanation for why hexadecanedioate is considered a risk factor for stroke.

## 5 Conclusion

In summary, this study is the first systematic MR analysis using genome-wide data to assess causal associations between serum metabolites and different cardiovascular diseases, providing preliminary evidence for the impact of circulating metabolic disorders on cardiovascular disease risk. Through IVW methods and multiple sensitivity analyses, we finalized the identification of 15 relevant metabolites, including 8 lipid metabolites and 2 amino acid metabolites. Our preliminary results indicate a potential for these metabolites to serve as circulating metabolic biomarkers for cardiovascular disease screening and prevention. However, further validation and research are required to conclusively establish their clinical utility. Furthermore, although these metabolites may provide candidate molecules for future mechanistic exploration and drug target selection, this view remains speculative until more in-depth studies are conducted.

However, it is worth noting that despite the results of this study, some limitations still exist. For example, the results of this study are mainly based on a specific cohort and population, and thus caution may be needed when generalizing to other populations. In addition, although this study assessed causal associations through MR analysis, there are certain assumptions and limitations associated with this approach, such as the selection of instrumental variables and multiplicity of validity. Therefore, in future studies, it is necessary to further expand the sample size to verify the applicability and stability of these metabolites in different populations, as well as to deeply explore their potential biological mechanisms and drug targets.

Overall, this study provides new perspectives and ideas for risk assessment and prevention of cardiovascular diseases, as well as important references and insights for future studies. We expect that more studies will focus on these metabolites in the future and explore their roles and mechanisms in cardiovascular diseases in depth, contributing more to the prevention and treatment of CVDs.

## Data Availability

The datasets presented in this study can be found in online repositories. The names of the repository/repositories and accession number(s) can be found in the article/Supplementary Material.

## References

[1] McGarrah Robert W Crown Scott B Guo-Fang Z Shah Svati H Newgard Christopher B. Cardiovascular metabolomics. Circ Res. (2018) 122(9):1238–58. 10.1161/CIRCRESAHA.117.311002 29700070 PMC6029726

[2] Kandy FC Jabir Nasimudeen R Kamal Mohammad A Nabil AM Damanhouri Ghazi A Waseem K Neopterin an immune biomarker of coronary artery disease and its association with other CAD markers. IUBMB Life. (2015) 67(6):453–9. 10.1002/iub.1390 26086324

[3] Nick T Denis K Lucy Wright F Adam T Radu H Aleksandra T Epidemiology of cardiovascular disease in Europe. Nat Rev Cardiol. (2022) 19(2):133–43. 10.1038/s41569-021-00607-3 34497402

[4] Anastasia P Grechko Andrey V Paolo P Myasoedova Veronika A Valentina A Orekhov Alexander N. The diabetes mellitus-atherosclerosis connection: the role of lipid and glucose metabolism and chronic inflammation. Int J Mol Sci. (2020) 21(5):1835. 10.3390/ijms21051835 32155866 PMC7084712

[5] Nan Z Xiaoting Y Xinxin Z Yanting S Fei G Baoqi Y Diabetes mellitus to accelerated atherosclerosis: shared cellular and molecular mechanisms in glucose and lipid metabolism. J Cardiovasc Transl Res. (2024) 17(1):133–52. 10.1007/s12265-023-10470-x 38091232

[6] Yaxin C Yufang X Hang C Zhengpei C Yongjie K Shuqing L Plasma metabolites and risk of seven cancers: a two-sample mendelian randomization study among European descendants. BMC Med. (2024) 22(1):90. 10.1186/s12916-024-03272-8 38433226 PMC10910673

[7] Wishart DS. Metabolomics for investigating physiological and pathophysiological processes. Physiol Rev. (2019) 99(4):1819–75. 10.1152/physrev.00035.2018 31434538

[8] Ashley vdS Stewart Isobel D Brigitte K Maik P Tahani A Friederike G Circulating metabolites modulated by diet are associated with depression. Mol Psychiatry. (2023) 28(9):3874–87. 10.1038/s41380-023-02180-2 37495887 PMC10730409

[9] Martino D Antonio N Bassareo Pier P Christian CD Giuseppe M. Metabolomic approach to redox and nitrosative reactions in cardiovascular diseases. Front Physiol. (2018) 9:672. 10.3389/fphys.2018.00672 29997515 PMC6031070

[10] Richmond RC Davey Smith G. Mendelian Randomization: concepts and scope. Cold Spring Harb Perspect Med. (2022) 12(1):a040501. 10.1101/cshperspect.a040501 34426474 PMC8725623

[11] Smith GD Ebrahim S. ‘Mendelian randomization': can genetic epidemiology contribute to understanding environmental determinants of disease? Int J Epidemiol. (2003) 32(1):1–22. 10.1093/ije/dyg070 12689998

[12] Birney E. Mendelian Randomization. Cold Spring Harb Perspect Med. (2022) 12(4):a041302. 10.1101/cshperspect.a041302 34872952 PMC9121891

[13] Boef AG Dekkers OM le Cessie S. Mendelian Randomization studies: a review of the approaches used and the quality of reporting. Int J Epidemiol. (2015) 44(2):496–511. 10.1093/ije/dyv071 25953784

[14] Burgess S Thompson SG. Bias in causal estimates from Mendelian randomization studies with weak instruments. Stat Med. (2011) 30(11):1312–23. 10.1002/sim.4197 21432888

[15] Marie V Chia-Yen C Benjamin N Ron D. Detection of widespread horizontal pleiotropy in causal relationships inferred from Mendelian randomization between complex traits and diseases. Nat Genet. (2018) 50(5):693–8. 10.1038/s41588-018-0099-7 29686387 PMC6083837

[16] Bowden J Davey Smith G Burgess S. Mendelian Randomization with invalid instruments: effect estimation and bias detection through Egger regression. Int J Epidemiol. (2015) 44(2):512–25. 10.1093/ije/dyv080 26050253 PMC4469799

[17] Yiheng C Tianyuan L Ulrika P-K Stewart Isobel D Guillaume B-L Tomoko N Genomic atlas of the plasma metabolome prioritizes metabolites implicated in human diseases. Nat Genet. (2023) 55(1):44–53. 10.1038/s41588-022-01270-1 36635386 PMC7614162

[18] Pierce BL Burgess S. Efficient design for Mendelian randomization studies: subsample and 2-sample instrumental variable estimators. Am J Epidemiol. (2013) 178(7):1177–84. 10.1093/aje/kwt084 23863760 PMC3783091

[19] Cuezva JM Chen G Alonso AM Isidoro A Misek DE Hanash SM The bioenergetic signature of lung adenocarcinomas is a molecular marker of cancer diagnosis and prognosis. Carcinogenesis. (2004) 25(7):1157–63. 10.1093/carcin/bgh113 14963017

[20] Hartwig FP Davey Smith G Bowden J. Robust inference in summary data mendelian randomization via the zero modal pleiotropy assumption. Int J Epidemiol. (2017) 46(6):1985–98. 10.1093/ije/dyx102 29040600 PMC5837715

[21] Yan L Hongyan L Shuting Y Bumei Z Xiaopei L Jiapei Y The effects of coagulation factors on the risk of endometriosis: a mendelian randomization study. BMC Med. (2023) 21(1):195. 10.1186/s12916-023-02881-z 37226166 PMC10210381

[22] Peter L Buring Julie E Lina B Hansson Göran K John D Sommer BM Atherosclerosis. Nat Rev Dis Primers. (2019) 5(1):56. 10.1038/s41572-019-0106-z 31420554

[23] Cortenbach KR Staal AH Schoffelen T Gorris MA Van der Woude LL Jansen AF Differences in local immune cell landscape between Q fever and atherosclerotic abdominal aortic aneurysms identified by multiplex immunohistochemistry. Elife. (2022) 11:e72486 10.7554/eLife.72486 35137689 PMC8871373

[24] Ploingarm P Malgorzata F Rachael F Marc D Willem SG Ehsan N Role of vascular smooth muscle cell phenotypic switching and calcification in aortic aneurysm formation. Arterioscler Thromb Vasc Biol. (2019) 39(7):1351–68. 10.1161/ATVBAHA.119.312787 31144989

[25] Alexander O Renate F Al-Allaf Faisal A Dieter B. Release of vesicular stomatitis virus spike protein G-pseudotyped lentivirus from the host cell is impaired upon low-density lipoprotein receptor overexpression. J Virol. (2015) 89(22):11723–6. 10.1128/JVI.01869-15 26339060 PMC4645664

[26] Andreas E Pontus D Marcus S Mollet Ines G Giuseppe A Helena G Sphingolipids contribute to human atherosclerotic plaque inflammation. Arterioscler Thromb Vasc Biol. (2016) 36(6):1132–40. 10.1161/ATVBAHA.116.305675 27055903

[27] Bao JX Su YT Cheng YP Zhang HJ Xie XP Chang YM. Vascular sphingolipids in physiological and pathological adaptation. Front Biosci (Landmark Ed). (2016) 21(6):1168–86. 10.2741/4448 27100498

[28] Christian D Kathrin A Julia D Katharina H Barbara M Christian S Metabolomic profiling of ascending thoracic aortic aneurysms and dissections—implications for pathophysiology and biomarker discovery. PLoS One. (2017) 12(5):e0176727. 10.1371/journal.pone.0176727 28467501 PMC5415060

[29] Yanyan C Hongmei J Xin Q Jinping W Meng Y Qiuhong G Circulating palmitoyl sphingomyelin is associated with cardiovascular disease in individuals with type 2 diabetes: findings from the China Da Qing diabetes study. Diabetes Care. (2022) 45(3):666–73. 10.2337/dc21-1520 35165706 PMC8918230

[30] Xin Q Hongmei J Jinping W Siyao H Meng Y Xinxing F Circulating palmitoyl sphingomyelin levels predict the 10-year increased risk of cardiovascular disease death in Chinese adults: findings from the Da Qing diabetes study. Cardiovasc Diabetol. (2024) 23(1):37. 10.1186/s12933-023-02116-8 38245731 PMC10800040

[31] Obinata H Hla T. Sphingosine 1-phosphate and inflammation. Int Immunol. (2019) 31(9):617–25. 10.1093/intimm/dxz037 31049553 PMC6939830

[32] Alessandro C Daniela L Klara K Graziamaria C Claudio dL Femminella Grazia D Sphingosine kinases and sphingosine 1-phosphate receptors: signaling and actions in the cardiovascular system. Front Pharmacol. (2017) 8:556. 10.3389/fphar.2017.00556 28878674 PMC5572949

[33] Eyal H Edgar A Levy Steven B Aziz Emad F. Pathway for the management of atrial fibrillation and atrial flutter. Crit Pathw Cardiol. (2017) 16(2):47–52. 10.1097/HPC.0000000000000109 28509703

[34] Smita N Irfan S Emir V Bloom Heather L Jones Dean P Dudley Samuel C. Statin therapy for the prevention of atrial fibrillation trial (SToP AF trial). J Cardiovasc Electrophysiol. (2011) 22(4):414–9. 10.1111/j.1540-8167.2010.01925.x 20946227 PMC3022954

[35] Yuan Z Liu T Huo X Wang H Wang J Xue L. Glutamine transporter SLC1A5 regulates ionizing radiation-derived oxidative damage and ferroptosis. Oxid Med Cell Longev. (2022) 2022:3403009. 10.1155/2022/3403009 36262284 PMC9576409

[36] Xin-Ya L Hai-Tao H Huan-Xin C Xiao-Cheng L Jun W Qin Y Preoperative plasma biomarkers associated with atrial fibrillation after coronary artery bypass surgery. J Thorac Cardiovasc Surg. (2021) 162(3):851–863.e3. 10.1016/j.jtcvs.2020.01.079 32197906

[37] Snipelisky D Chaudhry SP Stewart GC. The many faces of heart failure. Card Electrophysiol Clin. (2019) 11(1):11–20. 10.1016/j.ccep.2018.11.001 30717842

[38] Lopaschuk Gary D Karwi Qutuba G Rong T Wende Adam R Dale AE. Cardiac energy metabolism in heart failure. Circ Res. (2021) 128(10):1487–513. 10.1161/CIRCRESAHA.121.318241 33983836 PMC8136750

[39] Sun D Tiedt S Yu B Jian X Gottesman RF Mosley TH A prospective study of serum metabolites and risk of ischemic stroke. Neurology. (2019) 92(16):e1890–8. 10.1212/wnl.0000000000007279 30867269 PMC6550501

[40] Jacob B-T Shoshana B-S Jochanan B Yoelit M Rachel H Johannes P Synthesis and hypolipidemic and antidiabetogenic activities of beta,beta,beta’,beta'-tetrasubstituted, long-chain dioic acids. J Med Chem. (1989) 32(9):2072–84. 10.1021/jm00129a010 2788743

[41] Boehme AK Esenwa C Elkind MS. Stroke risk factors, genetics, and prevention. Circ Res. (2017) 120(3):472–95. 10.1161/CIRCRESAHA.116.308398 28154098 PMC5321635

[42] Cristina M Delyth G Gabi K Alharbi Nora HJ Md AS Martin M Metabolomic identification of a novel pathway of blood pressure regulation involving hexadecanedioate. Hypertension. (2015) 66(2):422–9. 10.1161/HYPERTENSIONAHA.115.05544 26034203 PMC4490909

